# Cancer, Clinical Trials, and Canada: Our Contribution to Worldwide Randomized Controlled Trials

**DOI:** 10.3390/curroncol28020143

**Published:** 2021-04-13

**Authors:** Shubham Sharma, J. Connor Wells, Wilma M. Hopman, Joseph C. Del Paggio, Bishal Gyawali, Nazik Hammad, Annette E. Hay, Christopher M. Booth

**Affiliations:** 1 Division of Cancer Care and Epidemiology, Queen’s University Cancer Research Institute, Kingston, ON K7L 3N6, Canada; shsharma@qmed.ca (S.S.); j.connorwells@gmail.com (J.C.W.); Bishal.Gyawali@kingstonhsc.ca (B.G.); 2 Department of Oncology, Queen’s University, Kingston, ON K7L 5P9, Canada; Nazik.Hammad@kingstonhsc.ca; 3 Department of Public Health Sciences, Queen’s University, Kingston, ON K7L 3N6, Canada; Wilma.Hopman@kingstonhsc.ca; 4 Department of Oncology, Northern Ontario School of Medicine, Thunder Bay, ON P3E 2C6, Canada; jdelpagg@lakeheadu.ca; 5 Department of Medicine, Queen’s University, Kingston, ON K7L 3N6, Canada; annette.hay@kingstonhsc.ca; 6 Canadian Cancer Trials Group, Queen’s University, Kingston, ON K7L 3N6, Canada

**Keywords:** cancer, clinical trials, research funding, Canada, high-income countries

## Abstract

Canada has a long tradition of leading practice-changing clinical trials in oncology. Here, we describe methodology, results, and interpretation of oncology RCTs with Canadian involvement compared to RCTs from other high-income countries (HICs). A literature search identified all RCTs evaluating anti-cancer therapies published 2014–2017. RCTs were classified based on the country affiliation of first authors. The study cohort included 636 HIC-led RCTs; 155 (24%) had Canadian authors. Three-quarters (112/155, 72%) of Canadian RCTs were conducted in the palliative setting, compared to two thirds (299/481, 62%) of RCTs from other HICs (*p* = 0.022). Canadian RCTs were more likely than those from other HICs to be supported by industry (85% vs. 69%, *p* < 0.001). The proportion of positive Canadian trials that met the ESMO-MCBS threshold for substantial clinical benefit was comparable to RCTs without Canadian authors (29% vs. 32%, *p* = 0.137). Thirteen percent (20/155) of all Canadian trials were affiliated with the Canadian Cancer Trials Group (CCTG). Canada plays a meaningful role in the global cancer research ecosystem but is overly reliant on industry support. The very low proportion of trials that identify a new treatment with substantial clinical benefit is worrisome. A renewed investment in cancer clinical trials is needed in Canada.

## 1. Introduction

Randomized controlled trials (RCTs) are the gold standard for evaluating the efficacy of new cancer therapies [1]. Since widespread adoption of the RCT in the 1970s, cancer clinical trials have evolved considerably. Work by our group and others have shown that RCTs have evolved over time to become larger, more likely to be industry-funded, with more multicenter and international involvement [2,3,4]. There has also been a marked shift to the use of surrogate primary endpoints [5].

While the majority of North American research output is driven by the United States (US), Canada’s cancer research spending is comparable to that of other high-income countries across Europe and Asia [6,7]. Like other parts of the world, most clinical research funding in Canada now comes from industry sources as it has become increasingly difficult for independent Canadian investigators to support the increasing costs of clinical trial infrastructure [8]. Several groups have demonstrated that the volume of research output is associated with allocation of research funds rather than burden of disease [9,10,11,12,13].

Prior studies have described global research spending by country (including that of Canada) [6,7]. Other studies have examined the allocation of cancer research funds within Canada across disease sites [9,10,11,12]. However, there is a lack of data that describes RCT output from Canada compared to other high-income countries (HICs). In this study, we describe cancer RCTs conducted in HICs during 2014–2017 and compare those with Canadian authors to those without any Canadian involvement. The objective was to compare and contrast methodology, results, and their interpretation of Canadian RCTs compared to other HICs. Results from this analysis can offer insights into how to increase the impact of clinical cancer research in Canada.

## 2. Materials and Methods

### 2.1. Study Design and Search Strategy

This retrospective cohort study was designed to identify cancer RCTs published globally between 2014 and 2017. The present report is a secondary analysis to understand Canada’s contribution to the global research ecosystem using other HICs as a comparator. The primary report of the global population including the electronic search strategy has been reported elsewhere [14]. A structured literature search was designed using PUBMED to identify phase III RCTs evaluating cancer-targeted therapies published during 2014–2017. Studies were included if they were: a phase III study, involved any type of cancer, and tested a cancer-directed therapy (systemic, radiation, or surgery). The final study population was restricted to RCTs led by HIC defined by the institutional affiliation of the first author.

### 2.2. Data Abstraction and Classification

All eligible studies were reviewed using a standardized data abstraction form to capture information regarding authorship, study design, and results. Data abstraction was performed independently by two authors (J.C.W. and S.S.). The senior author (C.M.B.) periodically performed random duplicate abstraction to ensure data abstraction was of high quality. At completion of data collection, 30 studies were randomly chosen for review; only 11/1020 variables (1%) were found to be discordant with the original assessment. Another author (J.D.P.) with extensive experience using the European Society of Medical Oncology-Magnitude of Clinical Benefit Results Scale (ESMO-MCBS) derived grades for all superiority studies of systemic therapy that met their primary endpoint. Studies were classified into country of origin based on the institutional affiliation of the first author.

### 2.3. Outcomes and Statistical Analysis

Descriptive results were generated for the full study cohort. Comparisons were made between studies that had any Canadian co-authors and those that were led by other HICs without any Canadian involvement. Journal impact factor (IF) was assigned using the impact factor from 2016 (regardless of year of publication), as reported by the Journal Citation Reports Impact Factor [15]. We compared the effect size (as measured through hazard ratio) of “positive” superiority RCTs between groups. Version 1.1 of the ESMO-MCBS was used to derive a grade based on the positive endpoint for systemic therapy [16]. Grades of A and B (curative setting) and 5 and 4 (palliative setting) were considered to be “substantial” benefit.

Statistical analysis was conducted using IBM SPSS version 26.0 for Windows (Armonk, New York, NY, USA, 2019). Outcomes were compared using the Pearson Chi Square or Fisher’s Exact test, and independent samples *t*-tests or the Mann–Whitney U as appropriate. *p* values less than 0.05 were considered significant; no adjustments for multiple comparisons were made.

## 3. Results

### 3.1. Results of the Search Strategy

The search strategy identified 2275 publications. As shown in Supplementary Figure S1, 1639 studies were not eligible for the present analysis. The final study cohort included 636 HIC-led RCTs of which 155 (24%) had Canadian authors; 31 RCTs were led by Canadian investigators (12 Canadian first author, 13 Canadian last author, and 6 had both).

### 3.2. Design Characteristics of Canadian RCTs

Among those RCTs in which Canadian investigators were middle authors, the most common leading countries were US (57%, 71/124), France (12%, 15/124), United Kingston (UK) (6%, 7/124), and Germany (4%, 5/124). Among HIC RCTs that did not have any Canadian author involvement, the most common countries for first authors were US (20%, 97/481), Japan (12%, 58/124), Germany (12%, 57/124), UK (10%, 49/124), and France (10%, 47/124).

**Figure 1:**
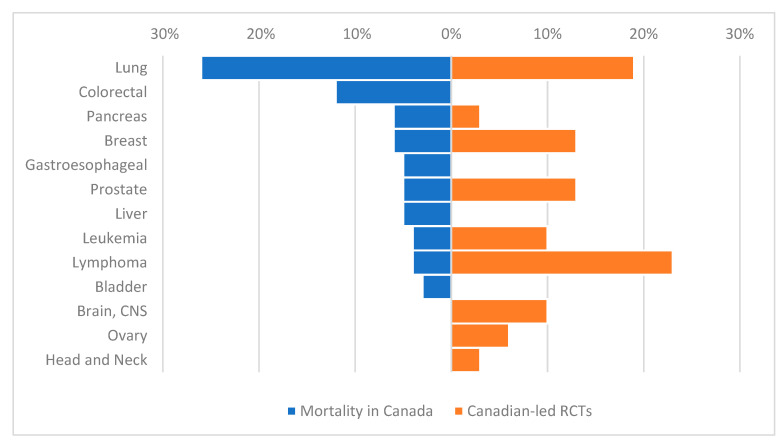
Ranking of top 10 cancers by proportion of all cancer deaths in Canada * and top 10 cancers by proportion of 31 Canadian-led randomized controlled trials published during 2014–2017. * From GLOBOCAN 2018 https://gco.iarc.fr/today/home (accessed on 19 October 2020).

Characteristics of the study cohort are presented in Table 1. The most common cancers enrolled in RCTs with Canadian authors were hematologic (21%, 32/155), genitourinary (17%, 26/155), breast (15%, 23/155), and lung (12%, 19/155). In comparison, the most common cancers in RCTs without Canadian authors were gastrointestinal (22%, 105/481), breast (19%, 92/481), hematologic (18%, 86/481), and lung (14% 65/481). The extent to which cancers studied in the 31 Canadian-led RCTs align with Canadian cancer mortality is shown in Figure 1. The proportion of Canadian-led RCTs relative to cancer mortality in Canada is substantially higher for lymphoma (23% RCTs, 4% deaths), breast (13% RCTs, 6% deaths), prostate (13% RCTs, 5% deaths), and leukemia (10% RCTs, 4% deaths); the proportion of RCTs compared to cancer mortality is substantially lower for lung (19% RCTs, 26% deaths), colorectal (<1% RCTs, 12% deaths), pancreas (3% RCTs, 6% deaths), and gastroesophageal (<1% RCTs, 5% deaths).

Three-quarters (112/155, 72%) of Canadian RCTs were conducted in the palliative setting, compared to two thirds (299/481, 62%) of HIC RCTs without Canadian authors (*p* = 0.022). The proportion of Canadian trials that tested new systemic therapies (88%, 137/155) or radiotherapy/surgery (11%, 17/155) was very similar to RCTs without Canadian involvement. The primary endpoint of Canadian RCTs was more likely to be overall survival [39% (61/155) vs. 29% (137/481), *p* = 0.011] compared to non-Canadian RCTs. One-third of RCTs in both groups [35% (54/155) and 33% (159/481)] have progression-free survival (PFS)/time to treatment failure (TTF) as primary endpoint. RCTs with Canadian authors were more likely than those without Canadian authors to be supported by industry [85% (132/155) vs. 69% (332/481), *p* < 0.001].

### 3.3. Results of Canadian RCTs

Details regarding the conduct and results of RCTs are shown in Table 2. The median sample size of Canadian RCTs was substantially larger than trials without Canadian authors [637 (IQR 410–991) vs. 419 (IQR 237–687)]. Canadian superiority trials were more likely to observe a statistically significant difference in favour of the experimental arm compared to trials from outside Canada [47% (70/150) vs. 39% (159/407), *p* = 0.021]. Among positive superiority RCTs, the observed effect size (HR 0.67 and HR 0.63, *p* = 0.571) and the proportion of trials that met ESMO-MCBS threshold for “substantial clinical benefit” [29% (14/49) and 32% (31/96), *p* = 0.137] was comparable between RCTs with and without Canadian authors. Thirteen percent (20/155) of trials with Canadian authors were affiliated with Canadian Cancer Trials Group (CCTG, formerly NCIC CTG). CCTG trials accounted for 23% (7/31) of RCTs led by Canadian authors and 11% (13/124) of RCTs with Canadian involvement but not first author.

### 3.4. Journal Impact Factor of Canadian RCTs

**Figure 2:**
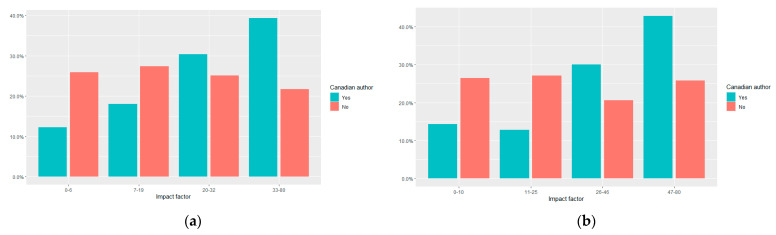
Journal impact factor of all oncology randomized controlled trials (RCTs) led by high-income countries published globally 2014–2017. RCTs are stratified by involvement of Canadian authors. Histogram bars reflect quartiles of all impact factors: (**a**) impact factor of all HIC RCTs for which an impact factor was available (*n* = 630); (**b**) impact factor for all positive superiority RCTs (*n* = 225).

The median IF for all HIC RCTs was 21 (IQR 7–34). RCTs with Canadian authors were published in journals with higher IF than those without Canadian authors [median IF 26 (IQR 18–36) vs. 18 (IQR 6–27), *p* < 0.001] (Figure 2). This observation persisted in a sensitivity analysis that considered only “positive” RCTs (median 36 (IQR 24–60) vs. IF 25 (IQR 11–48), *p* < 0.001).

## 4. Discussion

In this report, we provide an overview of oncology RCTs published during 2014–2017 with Canadian authors. Several important findings have emerged. First, Canadian investigators are co-authors on one-quarter of all oncology RCTs led by HICs. These trials are published in higher impact journals compared to RCTs without Canadian involvement. Second, the cancer RCTs that are led by Canadian authors do not match the burden of cancer in Canada. Third, most Canadian RCTs test new systemic therapies in the palliative setting and use surrogate outcomes (DFS and PFS) as the primary endpoint. Fourth, the vast majority (85%) of Canadian RCTs have industry funding; this is a higher proportion than RCTs from outside Canada. Finally, one-third of “positive” Canadian oncology RCTs identify a new treatment with “substantial clinical benefit”. This translates to only 13% of all Canadian clinical trials (the corresponding figure for non-Canadian RCTs is 12%). Our data suggest two fundamental threats to Canadian cancer trials: (1) the system is almost entirely reliant upon funding by industry (more so than other HICs); and (2) only a small minority of all Canadian RCTs identify a new treatment for patients that is associated with substantial clinical benefit.

Canada is a leader in cancer research and policy. It became one of the first countries to put forth a cancer plan in 2006 as recommended by the World Health Organization [17]. Since the inception of the cancer plan in 2006, Canada has seen reduced death rates from prostate and breast cancer, as well as increasing survival rates for breast, colorectal and lung cancers, along with most other cancer types [18,19]. Canada also has one of the highest survival rates from cancer among HICs with universal health care [20].

As reported by the Canadian Cancer Research Alliance (CCRA), there are important threats to the future of cancer clinical trials in Canada. Trials have become increasingly complex in terms of trial objectives and endpoints; time to trial activation has increased three-fold in a decade [4]. The cancer clinical trial landscape has also shifted from being academically driven to being mostly pharmaceutical industry-funded, in part due to increased trial costs. Although clinical care costs have remained relatively stable, costs of non-standard of care activities has increased substantially. These costs include flat fees for opening studies and costs related to trial coordination, analysis, and regulatory compliance [4]. Many clinical trial offices at hospitals have to balance their own budgets, ensuring all costs are secured for each individual trial. Industry studies provide substantially more per case funding that academic trials and it is therefore a pragmatic reality that most trials units need to ensure they open enough industry trials to “pay the bills”. This allows local investigators to also support academic and cooperative group Canadian trials in which they may have the opportunity to contribute to study design, collaborate with colleagues, and serve as co-authors.

The 2011 report by CCRA highlighted the main goals of cancer clinical trials, which consist of achieving better cancer control, increasing survival, and improving patient quality of life. Their vision was to enhance efficiencies and deploy resources more strategically. Key recommendations included: creation of pan-Canadian infrastructure to provide stable funding to support cancer clinical trials; engaging with key stakeholders such as Health Canada to streamline the clinical regulatory framework; developing reciprocity in research ethics boards to prevent duplicate efforts while complementing content knowledge; and reviewing routine practices in trial development that add time and cost but little value [4].

For five decades the United States National Cancer Institute (NCI) has led cutting edge research. Canadians benefit directly from the NCI US, not least through its investment in the Canadian Cancer Trials Group, the only non-American partner of the US National Clinical Trials Network [21]. In the United Kingdom, government funding directly supports clinical trial networks, embedding clinical research as a core component of health care delivery through the National Health Service. In Canada, no such comprehensive program exists to support academic clinical trials.

To exemplify the Canadian context, we undertook a detailed review of the 32 hematology randomized trials included within our study cohort; we also included one additional CCTG trial [22] in this detailed review which was not identified on the original literature search. Seven (21%) of these trials were led by an academic cooperative group (3 CCTG, 3 Children’s Oncology Group, 1 Dana Farber Cancer Institute); 6/7 of these cooperative group trials were funded by the US NCI. The remaining one [22], was funded primarily by the Canadian Cancer Society Research Institute with partial support in the form of drug supply from 2 pharmaceutical companies. The remaining 26/33 (79%) of hematology trials conducted in Canada were industry sponsored and led, demonstrating that industry sees Canada as a place to conduct high-quality clinical research, including studies that will lead to new regulatory approvals.

Our data illustrate a disconnect between the cancer burden in Canada and the trials that are led by Canadian oncologists. Our group has previously identified that research funding and clinical trial output in Canada is not proportional to the burden of disease [9]. The low success rate of trials (i.e., 13% of all trials identify a major treatment advance) speaks to opportunities to improve trial rationale and design. These collective observations (together with the fact that almost all trials rely on industry support) highlights the need for a renewed strategy and greater investment in Canadian cancer clinical trials.

Finally, our study highlights the need for strategic government investment in clinical trial infrastructure and research. Pharmaceutical companies and academics are both driven to improves outcomes for people with cancer. Both can do it well, as evidenced by the recent impressive speed in developing COVID19 vaccines, and the practice changing research led by both academics and industry which forms the basis of this manuscript. However, their secondary goals can be divergent [23]. Industry must answer to shareholders and generate profit. While industry-led trials play an important role in advancing patient care, academic cooperative groups such as the CCTG are committed to testing new treatments that not only improve patient outcomes but may also reduce toxicity, treatment intensity, and system-level costs associated with cancer care. Given the concerns that industry is now directing the vast majority of clinical trials in oncology, we believe it is essential for Canada to invest to ensure that the research agenda is dictated by the needs of patients and not industry. This will require increased government investment in cancer research infrastructure to support high-impact clinical trials.

Canada has all the components necessary to conduct high quality clinical trial research: (i) patients willing to put themselves forward and contribute to scientific discovery, (ii) physicians/clinician scientists committed to advancing science and healthcare, (iii) research sites with trained staff to deliver complex care and report data accurately and quickly, (iv) a national academic cooperative group with over 40 years experience in over 500 trials involving more than 85,000 patients. Academic cooperative groups conduct high quality clinical trials, specifically designed to minimize bias and inform policy and practice. Within Canada, research funding for academic clinical trials can and has been successfully secured from the Canadian Institutes for Health Research (CIHR); these highly competitive competitions have success rates in the range of 10%. Research funding for clinical trials can also be obtained from charitable organizations which represent important contributions; generous members of the public can only support a fraction of the important questions on improving cancer treatment. Thus, academic groups commonly end up aligning with industry on questions of mutual interest, those testing drugs hypothesized to improve the lives of patients with cancer, usually while also holding potential to expand the market for pharmaceutical partners. Industry is primarily interested in studies of new cancer medicines. There is a huge unmet need for other studies such as in radiotherapy, surgery, palliative care, psychosocial oncology and repurposing of established drugs. The current system which favours research aligning with industry goals will identify more costly therapies resulting in increasing demands of an already stretched health care budget. Allocation of a proportion of health care spending to support clinical trial infrastructure and academic research would enable studies intended to reduce health care spending (i.e., a modest investment now could result in cost savings within the next 5–10 years).

Our results should be considered in light of methodologic limitations. Although we focused exclusively on phase III RCTs, we recognize and appreciate that there are forms of research contribution (i.e., translational science, early phase trials, systematic review/meta-analysis, observational studies, policy analysis) which also contribute to improvements in cancer care and outcomes. Our study does not offer insight into Canada’s contribution in these fields. RCTs in our study were classified based on authorship which may not necessarily reflect where patients were enrolled. There were a small number of trials that were led by Canadian authors (*n* = 31); however, this sample size was not large enough to allow for detailed analyses in this group. As a result, we were unable to differentiate studies that were led by Canada from those that were led by other HICs with Canadian involvement. This limits our ability to specifically evaluate the research agenda of Canadian-led trials. Our literature search inevitably would have missed some RCT reports although we do not think this would substantially alter our key findings. Our dataset also did not distinguish trials with academic sponsors with industry funding support, from those which were funded and sponsored by industry. Finally, we did not evaluate the methodologic rigor of the RCTs nor did we measure the impact of the trials in change practice, guidelines, or outcomes.

## 5. Conclusions

In summary, Canada plays a meaningful role in the global RCT ecosystem. Canadian RCTs are heavily dependent on funding from the pharmaceutical industry and primarily test new systemic therapies in the palliative context. Only one third of positive Canadian trials (and 13% of all trials) identify a new treatment that is associated with substantial clinical benefit; urgent efforts to improve on this are warranted. It is notable that only 14% of Canadian trials are conducted without industry funding. These data highlight the need for more investment in cancer clinical trials from the government and philanthropic sectors to ensure that Canadian RCTs address diseases and questions that are most likely to improve outcomes for patients in Canada and beyond.

## Figures and Tables

**Table 1:** Characteristics of all high-income oncology randomized controlled trials published globally 2014–2017.

	All HIC RCTs	Author Involvement		
	*n* = 636	Canada (*n* = 155)	Other HIC (*n* = 481)	*p*-Value
	*n* (%)	*n* (%)		
Disease site				
Breast	115 (18%)	23 (15%)	92 (19%)	<0.001
Lung	84 (13%)	19 (12%)	65 (14%)	
GI	116 (18%)	11 (7%)	105 (22%)	
Head and Neck	24 (4%)	5 (3%)	19 (4%)	
Heme	118 (19%)	32 (21%)	86 (18%)	
GU	64 (10%)	26 (17%)	38 (8%)	
Gyne	35 (6%)	9 (6%)	26 (5%)	
Skin	32 (5%)	12 (8%)	20 (4%)	
CNS/Brain	20 (3%)	10 (6%)	10 (2%)	
Other	28 (4%)	8 (5%)	20 (4%)	
Treatment intent ^1^				
Palliative	411 (65%)	112 (72%)	299 (62%)	0.066
Curative	61 (10%)	13 (8%)	48 (10%)	
Neoadjuvant/adjuvant	164 (26%)	30 (19%)	134 (28%)	
Experimental arm				
Systemic	556 (87%)	137 (88%)	419 (87%)	0.671
Radiation	34 (5%)	5 (3%)	29 (6%)	
Surgery	15 (2%)	4 (3%)	11 (2%)	
Combination ^2^	26 (4%)	8 (5%)	18 (4%)	
Other ^3^	5 (1%)	1 (1%)	4 (1%)	
Control arm				
Active therapy	525 (83%)	123 (79%)	402 (84%)	<0.001
Placebo	63 (10%)	28 (18%)	35 (7%)	
Observation/BSC	48 (8%)	4 (3%)	44 (9%)	
Primary endpoint				
OS	198 (31%)	61 (39%)	137 (29%)	0.057
DFS/EFS/RFS	142 (22%)	28 (18%)	114 (24%)	
PFS/TTF	213 (34%)	54 (35%)	159 (33%)	
QOL/toxicity	20 (3%)	3 (2%)	17 (4%)	
RR	35 (5%)	5 (3%)	30 (6%)	
Other	28 (4%)	4 (3%)	24 (5%)	
Industry funding				
Yes	464 (73%)	132 (85%)	332 (69%)	<0.001
No	149 (23%)	21 (14%)	128 (27%)	
Unstated	23 (4%)	2 (1%)	21 (4%)	

^1^ Column total does not add to 636 due to missing data, or due to rounding. ^2^ Combined experimental arms include systemic-RT (*n* = 22), systemic-surgical (*n* = 3), surgery-radiation (*n* = 1), ^3^ Other experimental interventions included hyperthermia plus RT (*n* = 2), photodynamic therapy, stem cell transplant, tumor treating field (*n* = 1 each).

**Table 2:** Results of all oncology randomized controlled trials published by high-income countries during 2014–2017 (*n* = 636).

	All HIC RCTs	Author Involvement		*p*-Value
	*n* = 636	Canada *n* = 155	Other HIC *n* = 481	
Sample size				
Median (IQR)	474 (262–743)	637 (410–991)	419 (237–687)	<0.001
*p* < 0.05 for primary endpoint ^1^	*n* = 557	*n* = 150	*n* = 407	0.021
Yes	229 (41%)	70 (47%)	159 (39%)	
No	328 (59%)	80 (53%)	248 (61%)	
HR for + superiority RCTs ^2^				
Median (IQR)	0.65 (0.52–0.75)	0.67 (0.51–0.77)	0.63 (0.52–0.75)	0.571
ESMO-MCBS grade ^3^	*n* = 145	*n* = 49	*n* = 96	0.137
Substantial benefit (A,B,4,5)	45 (31%)	14 (29%)	31 (32%)	
Not substantial benefit (C,1,2,3)	100 (69%)	35 (71%)	65 (68%)	

^1^ Only reported for *n* = 559 superiority trials, ^2^ Only reported for *n* = 205 positive superiority trials, ^3^ Only reported for 145/205 positive superiority trials.

## Data Availability

There is no publicly archived dataset available. The authors utilized publicly available information from published RCTs to create a database for this study.

## Supplementary Materials

The following are available online at https://www.mdpi.com/article/10.3390/curroncol28020143/s1, Figure S1: Results of search strategy for all oncology randomized controlled trials conducted during 2014–2017.
