# Supplementary material for: Cancer, Clinical Trials, and Canada: Our Contribution to Worldwide Randomized Controlled Trials
Source: Curr Oncol. 2021 Apr 13;28(2):1518–27. doi: 10.3390/curroncol28020143 (PMC8167552; doi:10.3390/curroncol28020143)
Supplement: Supplementary file 1 [file curroncol-28-00143-s001.pdf]

Supplementary Materials: Cancer, Clinical Trials, and Canada: Our Contribution to Worldwide Randomized Controlled Trials

Shubham Sharma, J. Connor Wells, Wilma M. Hopman, Joseph C. Del Paggio, Bishal Gyawali, Nazik Hammad, Annette E. Hay, Christopher M. Booth

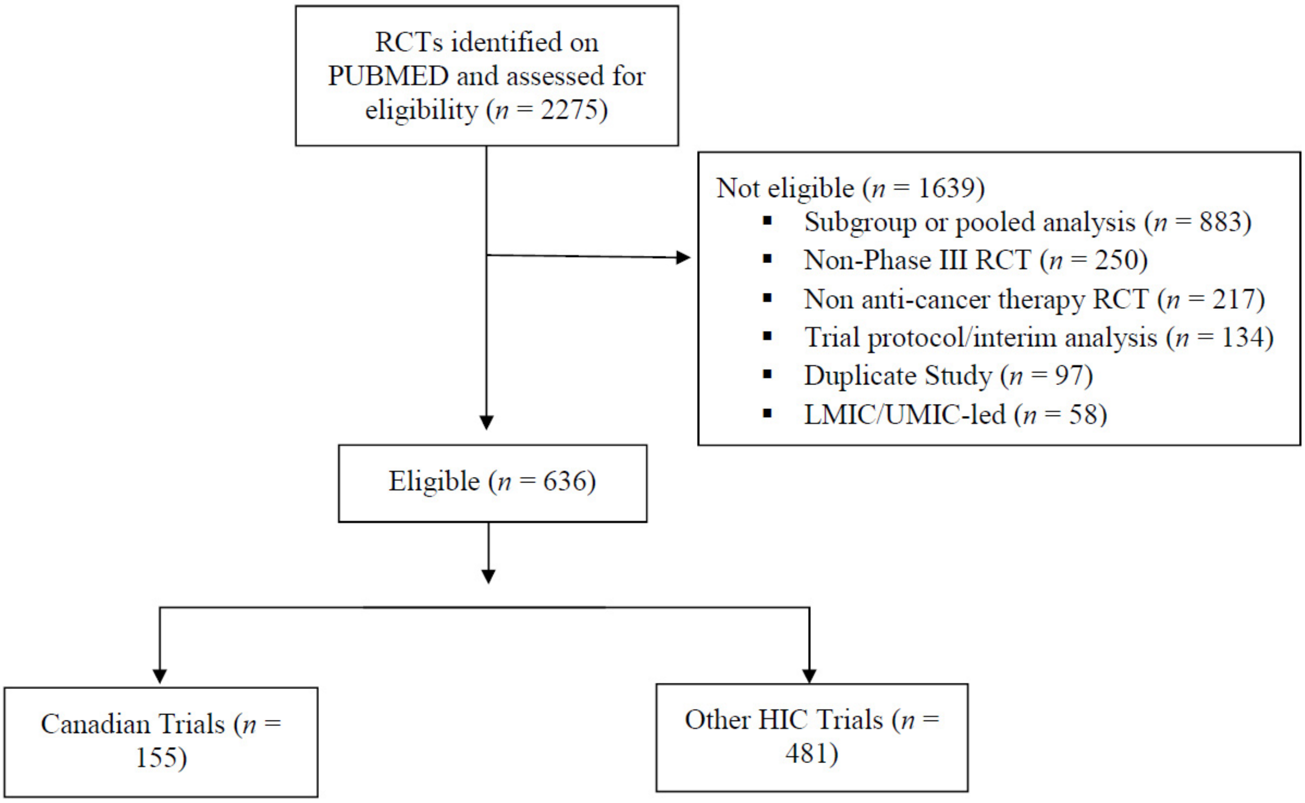

Figure S1. Results of search strategy for all oncology randomized controlled trials conducted during 2014–2017.
